# Risk factors associated with glycated hemoglobin A1c trajectories progressing to type 2 diabetes

**DOI:** 10.1080/07853890.2022.2164347

**Published:** 2023-01-09

**Authors:** Alexandra Halalau, Sujoy Roy, Arpitha Hegde, Sumesh Khanal, Emily Langnas, Maidah Raja, Ramin Homayouni

**Affiliations:** a Department of Internal Medicine, Beaumont Hospital, Royal Oak, MI, USA; b Oakland University William Beaumont School of Medicine, Rochester, MI, USA; c Department of Foundational Medical Studies, Oakland University William Beaumont School of Medicine, Rochester, MI, USA; d Department of Electrical and Computer Engineering, Oakland University, Rochester, MI, USA; e Department of Internal Medicine, Rochester General Hospital, Rochester, NY, USA

**Keywords:** Prediabetes, hemoglobin A1c, trajectory, type 2 diabetes mellitus, risk factors

## Abstract

**Background and objective:**

The notion of prediabetes, defined by the ADA as glycated hemoglobin A1c (HbA1c) of 5.7–6.4%, implies increased vascular inflammatory and immunologic processes and higher risk for developing diabetes mellitus and major cardiovascular events. We aimed to determine the risk factors associated with rapid progression of normal and prediabetes patients to type 2 diabetes mellitus (T2DM).

**Methods:**

Retrospective cohort study in a single 8-hospital health system in southeast Michigan, between 2006 and 2020. All patients with HbA1c <6.5% at baseline and at least 2 other HbA1c measurements were clustered in five trajectories encompassing more than 95% of the study population. Multivariate linear regression analysis was performed to examine the association of demographic and comorbidities with HbA1c trajectories progressing to diabetes.

**Results:**

A total of 5,347 prediabetic patients were clustered based on their HbA1c progression (C1: 4,853, C2: 253, C66: 102, C12: 85, C68: 54). The largest cluster (C1) had a baseline median HbA1c value of 6.0% and exhibited stable HbA1c levels in prediabetic range across all subsequent years. The smallest cluster (C68) had the lowest median baseline HbA1c value and also remained stable across subsequent years. The proportion of normal HbA1c in each of the pre-diabetic trajectories ranged from 0 to 12.7%, whereas 81.5% of the reference cluster (C68) were normal HbA1c at baseline. The C2 (steady rising) trajectory was significantly associated with BMI (adj OR 1.10, 95%CI 1.03–1.17), and family history of DM (adj OR 2.75, 95%CI 1.32–5.74). With respect to the late rising trajectories, baseline BMI was significantly associated with both C66 and C12 trajectory (adj OR 1.10, 95%CI 1.03–1.18) and (adj OR 1.13, 95%CI 1.05–1.23) respectively, whereas, the C12 trajectory was also significantly associated with age (adj OR 1.62, 95%CI 1.04–2.53) and history of MACE (adj OR 3.20, 95%CI 1.14–8.93).

**Conclusions:**

We suggest that perhaps a more aggressive preventative approach should be considered in patients with a family history of T2DM who have high BMI and year-to-year increase in HbA1c, whether they have normal hemoglobin A1c or they have prediabetes.

**KEY MESSAGES**
- Progression to diabetes from normal or prediabetic hemoglobin A1c within four years is associated with baseline BMI.
- A steady rise in HbA1c during a four-year period is associated with age and family history of T2DM, whereas age and personal history of MACE are associated with a rapid rise in HbA1c.
- A more aggressive preventative approach should be considered in patients with a family history of T2DM who have high BMI and year-to-year increase in HbA1c.

## Introduction

It is well known that type 2 diabetes mellitus (T2DM) is associated with cardiovascular disease and a variety of other co-morbidities [1]. Recent studies utilizing large-scale data from electronic medical records (EMR) have shown that different trajectories of HbA1c in patients with newly diagnosed diabetes mellitus have different risks of complications and mortality [2–7]. For example, moderately increasing HbA1c is associated with renal disease progression in patients with T2DM [8].

Prediabetes affects one in three Americans [1]. American Diabetes Association (ADA) defines prediabetes as a state of elevated blood glucose (fasting plasma glucose of 100–125 mg/dl and/or 2-hour plasma glucose after oral glucose challenge of 140–199 mg/dl and/or hemoglobin A1C of 5.7–6.4%) that doesn’t meet the criteria for overt diabetes [9]. Prediabetes increases the risk of progression to overt diabetes, with annual rate of progression estimated at 5–15% [10]. Meta-analysis of prospective cohort studies from general population showed that prediabetes was associated with increased risk of cardiovascular disease [11]. Also, a large retrospective study that included 119,271 patients showed that prediabetes is an independent risk factor for major adverse cardiovascular events [12].

The time course of progression from prediabetes to diabetes differs across populations. While some individuals remain stable at prediabetes ranges of HbA1c or fasting plasma glucose levels for many years, others rapidly develop diabetes [13]. Recent studies have investigated the association between different trajectories and risk of health complications or mortality. Interestingly, some studies attribute increased risk of complications and mortality to the progression from prediabetes to diabetes [14–16], while others suggest that these risks are independent of progression to diabetes [17,18].

In this retrospective observational cohort study, we used data from the EMR of a large health system to analyze common trajectories of HbA1c values in patients with prediabetes and to identify risk factors that are associated with different trajectories of HbA1c.

## Methods

### Study design & setting

This retrospective observational cohort study included data from the electronic health records from September 2007 to January 2020, from the Beaumont Health System, which is comprised of eight hospitals and 167 associated outpatient locations in the Detroit metropolitan area. All data were obtained through automated queries using Toad Data Point multi-platform database query tool and EPIC Clarity data warehouse (EPIC System, Verona, WI, USA). The study was approved by the Beaumont Health Institutional Review Board (IRB # 2019-281).

### Participants

The initial cohort included all patients with at least three valid HbA1c values (within 2 and 19%). For each patient, only the highest HbA1c was used per calendar year when multiple HbA1c values existed in a given year. Next, the all-time highest HbA1c was selected as the index date and the prior four years data was used for trajectory analysis, such that the baseline year was four years prior to the index year. The cohort was further refined by the following criteria: (1) Baseline HbA1c below 6.5, (2) two or fewer missing HbA1c values between baseline and index years, (3) no diagnosis of Diabetes Mellitus (DM) at baseline or prior years, (4) age greater than 18, and (5) valid values for date of birth and gender.

### Variables

HbA1c values were categorized as follows: (1) Normal, HbA1c <5.7%, (2) prediabetes, HbA1c ≥5.7% and ≤6.4%, and (3) diabetes, HbA1c ≥6.5% [9]. The variables assessed were baseline characteristics of age, gender, race, ethnicity, body mass index (BMI), co-morbidities such as hypertension, hyperlipidemia, peripheral vascular disease, anemia, cancer, chronic kidney disease (CKD), chronic obstructive pulmonary disease (COPD), obstructive sleep apnea (OSA), family history of myocardial infarction (MI), coronary artery disease (CAD), DM and stroke as well as the presence of major adverse cardiovascular events (MACE) outcomes. The ICD-10-CM codes corresponding to the chronic conditions were defined by Center for Medicaid and Medicaid Services (Chronic Conditions Data Warehouse, 2021 version). MACE included any of the following conditions: non-ST and ST-elevation myocardial infarction, unstable angina, coronary artery disease, heart failure, ischemic stroke, percutaneous coronary intervention, coronary artery bypass graft, and all-cause mortality. MACE was categorized per individual as either being present (≥1 event) or absent (no events) during the study time frame. To decrease information bias, both medical histories and problem lists were queried to account for differences in physician documentation.

### Statistical methods

Using Python scripts, raw data from the reports were merged into a master table, where each row represented a unique patient and columns included each variable represented as either a continuous or categorical value. Mean-Shift Clustering was performed on the final set of patients that met the inclusion criteria described above. Prior to clustering, all missing values were imputed using the K-nearest neighbor method with *K* = 2. A total of 68 clusters were obtained. Only five clusters, containing ≥50 patients each, were considered for further analyses.

To test if any of the baseline variables were significantly different between the five trajectories, Kruskal–Wallis tests were performed for continuous variables and Chi-square tests were performed for categorical variables, where appropriate (expected frequency in at least 80% of cells ≥5), otherwise Fisher’s exact tests were used.

To determine which baseline variables were associated with specific trajectories, we performed pair-wise multivariate logistic regression analysis where each cluster was compared to C68 as the reference cluster. The independent variables in the model included age, BMI, gender, SBP, family history of diabetes mellitus (Family_DM), family history of stroke (Family_stroke) and major adverse cardiac events (MACE) at baseline or prior years. A step-down logistic regression analysis was performed by dropping the least significant variable at each step until only variables with a *p*-value <0.05 remained in the final model.

## Results

**Figure 1.**
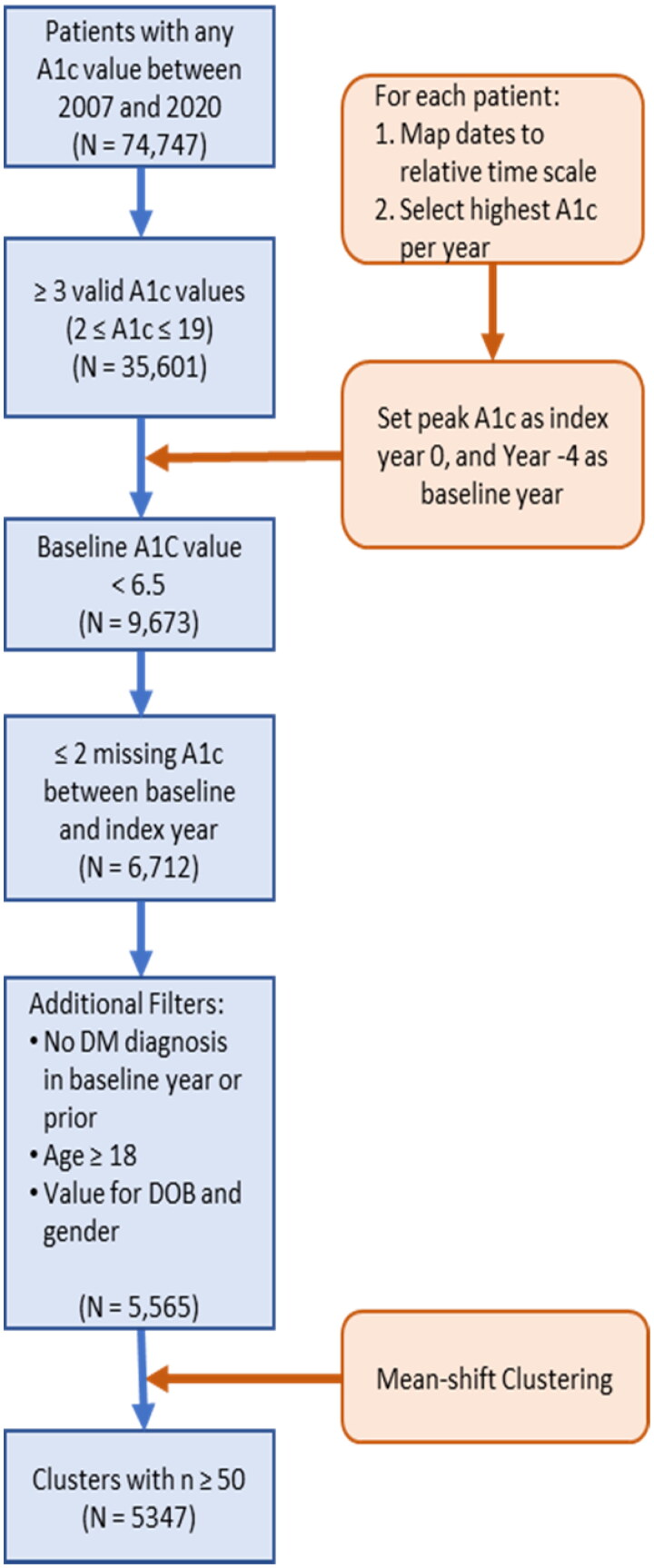
Workflow diagram of cohort selection criteria and HbA1c trajectory determination. A1c: hemoglobin A1c; DM: diabetes mellitus; DOB: date of birth.

Based on the inclusion and exclusion criteria outlined in Figure 1, the final cohort for this study included 5,565 patients. All individuals in the study had HbA1c values at baseline and at the four year follow up time (index year). A total of 2,546 individuals had HbA1c values for all five consecutive years, while 1,754 and 1,265 individuals were missing one and two HbA1c values during this time frame, respectively.

**Figure 2.**
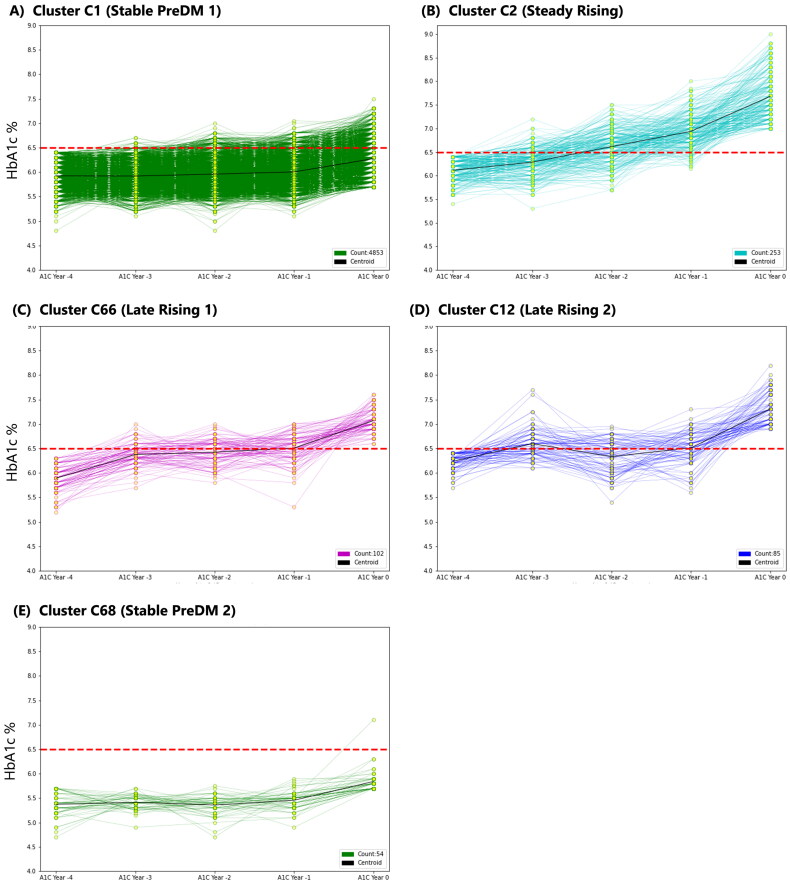
Top five HbA1c trajectories produced by mean-shift clustering. The clusters are presented in order of sample size.

Mean-Shift clustering using five consecutive years of HbA1c values (with imputation) resulted in five distinct trajectories with at least 50 patients, (C1: 4,853, C2: 253, C66: 102, C12: 85, C68: 54), amounting to a total of 5,347 patients (>95% of the study cohort). The remaining 218 patients were spread across multiple smaller clusters, which were not used for subsequent statistical analysis due to their small sample sizes. The largest cluster (C1, *n* = 4853) had a baseline median HbA1c value of 6.0% and exhibited stable HbA1c levels in prediabetic range across all subsequent years. The smallest cluster (C68, *n* = 54) had the lowest median baseline HbA1c value and also remained stable across subsequent years. The other three clusters exhibited an increase in HbA1c values across the five-year period. Cluster C2 (*n* = 253) exhibited a steady rise in HbA1c levels over the five-year period, starting at a median HbA1c value of 6.0% at baseline and ending at 7.5% by the index year. The final two clusters (C66 and C12) exhibited stable HbA1c values near 6.5% in the first four years with a rapid rise in HbA1c during the final year (Figure 2).

The baseline characteristics of the patients in each of the trajectory clusters are provided in Table 1. The proportion of males across these clusters varied significantly (*p* < 0.013, Chi-square test), with C66 having the lowest proportion of males (42.2%) and C12 having the highest proportion of males (55.3%). The median age was also significantly (*p* < 0.001, Kruskal–Wallis) different across the clusters, with C68 having the lowest median age (58.3, IQR 17.9) and C12 with the highest median age (66.2, IQR 15.3). Median BMI was significantly (*p* < 0.0002) different across the clusters, with C68 having the lowest median BMI (29.2, IQR 10.3) compared to C66 and C12 with (35.1, IQR 8.4) and (33.55, IQR 8.2), respectively. Median systolic blood pressure was significantly (*p* < 0.0015) different across the clusters, with C68 having (133, IQR 28 mmHg) and C12 having (148, IQR 28 mmHg). There were also significant differences across the clusters for family history of diabetes mellitus (*p* < 0.012, Chi-Square), as well as personal history of ischemic heart disease (IHD, *p* < 0.025, Chi-Square), major adverse cardiovascular event (MACE, *p* < 0.022) and obstructive sleep apnea (OSA, *p* < 0.41) at baseline or prior years. Since the number of observations for OSA were few, we did not include OSA in further analysis. In addition, since IHD is included in the aggregate variable MACE, we did not include IHD in further analysis.

**Table 1.** Baseline characteristics of the patients belonging to five distinct Hb1Ac trajectories.

	C1	C68	C2	C66	C12	*p*-Value
	Stable PreDM	Stable normal	Steady rising	Late rising 1	Late rising 2	
	*N* = 4853	*N* = 54	*N* = 253	*N* = 102	*N* = 85	
Age, *N*	63.2 (15.7)	58.3 (17.9)	61.0 (19.0)	61.2 (14.1)	66.2 (15.3)	**0.0011**
Median (IQR)						
Males (%)	2094 (43.2%)	24 (44.4%)	132 (52.2%)	43 (42.2%)	47 (55.3%)	**0.013**
Race	*N* = 4783	*N* = 54	*N* = 246	*N* = 102	*N* = 84	0.61
Asian	255 (5.4)	3 (5.6)	15 (6.1)	4 (3.9)	2 (2.4)	
Black	691 (14.5)	6 (11.1)	31 (12.6)	9 (8.8)	8 (9.5)	
White	3488 (73.2)	41 (75.9)	182 (74.0)	81 (79.4)	71 (84.5)	
Other	329 (6.9)	4 (7.4)	18 (7.3)	8 (7.8)	3 (3.6)	
HbA1c <5.7 (%)	487 (10.0)	44 (81.5)	9 (3.6)	13 (12.7)	0 (0.0)	**0.0001**
BMI, *N*	*N* = 3374	*N* = 40	*N* = 167	*N* = 69	*N* = 61	**0.0002**
Median (IQR)	31.9 (9.0)	29.2 (10.3)	33.1 (9.0)	35.1 (8.4)	33.5 (8.2)	
SBP, *N*	*N* = 3301	*N* = 40	*N* = 166	*N* = 67	*N* = 61	**0.0015**
Median (IQR)	140 (20)	133 (28)	140 (20)	140 (24)	148 (23)	
DBP, *N*	*N* = 3302	*N* = 40	*N* = 166	*N* = 67	*N* = 61	0.2584
Median (IQR)	82 (12)	81 (10)	84 (14)	85 (14)	85 (14)	
Family CD (%)	978/3986 (24.5)	13/50 (26.0)	56/210 (26.7)	28/90 (31.1)	13/66 (19.7)	0.49
Family DM (%)	2078/3986 (52.1)	19/50 (38.0)	131/210 (62.4)	49/90 (54.4)	34/66 (51.5)	**0.012**
Family MI (%)	725/3986 (18.2)	8/50 (16.0)	49/210 (23.3)	12/90 (13.3)	12/66 (18.2)	0.26
Family Stroke (%)	1161/3986 (29.1)	11/50 (22.0)	67/210 (31.9)	37/90 (41.1)	15/66 (22.7)	0.051
AFib (%)	135 (0.7)	3 (5.6)	9 (3.6)	6 (5.9)	3 (3.5)	0.16
Angina (%)	82 (1.7)	0	5 (2.0)	2 (2.0)	4 (4.7)	0.25
CAD (%)	258 (5.3)	2 (3.7)	11 (4.4)	7 (6.9)	9 (10.6)	0.21
Cancer (%)	170 (3.5)	0	13 (5.1)	6 (5.9)	2 (2.4)	0.22
CKD (%)	199 (4.1)	1 (1.9)	14 (5.5)	8 (7.8)	6 (7.1)	0.13
COPD (%)	172 (3.5)	3 (5.6)	6 (2.4)	5 (4.9)	1 (1.2)	0.42
DYS (%)	206 (4.2)	0	13 (5.1)	6 (5.9)	4 (4.7)	0.41
HF (%)	57 (1.2)	2 (3.7)	5 (2.0)	2 (2.0)	1 (1.2)	0.17
HTN (%)	1160 (23.9)	10 (18.5)	46 (18.2)	21 (20.6)	17 (20.0)	0.18
IHD (%)	342 (7.1)	2 (3.7)	15 (5.9)	10 (9.8)	13 (15.3)	**0.025**
MACE (%)	1309 (27.0)	11 (20.4)	71 (28.1)	29 (28.4)	36 (42.4)	**0.022**
MI (%)	66 (1.4)	0	4 (1.6)	3 (2.9)	3 (3.5)	0.20
OSA (%)	280 (5.8)	2 (3.7)	10 (4.0)	13 (12.8)	3 (3.5)	**0.041**
PVD (%)	88 (1.8)	0	6 (2.4)	1 (1.0)	3 (3.5)	0.54
S/TIA (%)	106 (2.2)	2 (3.7)	1 (0.4)	3 (2.9)	2 (2.4)	0.15

*p*-Value were determined using Kruskal–Wallis tests for continuous variables and Chi-square tests for categorical variables. p-Values below 0.05 are bolded.  (BMI: body mass index; SBP: systolic blood pressure; DBP: diastolic blood pressure; Family CD: family history of coronary disease; Family DM: family history of diabetes mellitus; Family MI: family history of myocardial infarction; CAD: coronary artery disease; CKD: chronic kidney disease; COPD: chronic obstructive pulmonary disease; DYS: dyspnea; HF: heart failure; HTN: hypertension; IHD: ischemic heart disease; MACE: major adverse cardiovascular events; OSA: obstructive sleep apnea; PVD: peripheral vascular disease; S/TIA: stroke/transient ischemic attack).

The results from two different multi-variate linear regression models are shown in Table 2. The first model included independent variables that were significantly different among the trajectories by univariate analysis (Table 1). The second model included variables after a stepwise backward selection. Based on the final model (Model 2), the C2 (steady rising) trajectory was significantly associated with BMI (adj OR 1.10, 95% Wald CI 1.03–1.17), and family history of DM (adj OR 2.75, 95% Wald CI 1.32–5.74). With respect to the late rising trajectories, baseline BMI was significantly associated with both C66 and C12 trajectory (adj OR 1.10, 95% Wald CI 1.03–1.18) and (adj OR 1.13, 95% Wald CI 1.05–1.23), respectively. Whereas, the C12 trajectory was also significantly associated with age (adj OR 1.62, 95% Wald CI 1.04–2.53), BMI (adj OR 1.13, 95% Wald CI 1.05–1.23), and history of MACE (adj OR 3.20, 95% Wald CI 1.14–8.93).

**Table 2.** Adjusted odds ratios of baseline risk factors associated with three trajectories ending with HbA1c above 6.5 in the final index year (C2, C66, and C12) compared to a reference trajectory with stable HbA1c below 6.5 (C68).

	Steady rising HbA1c (C2)		Late rising HbA1c (C66)		Late rising HbA1c (C12)	
	Model 1*	Model 2	Model 1	Model 2	Model 1	Model 2
Age	1.27	**1.40**	1.32		1.49	**1.62**
	[0.90–1.77]	**[1.04–1.88]**	[0.83–2.08]		[0.89–2.1]	**[1.04–2.53**
BMI	1.11**	**1.10**	**1.17**	**1.10**	**1.17**	**1.13**
	**[1.04–1.18]**	**[1.03–1.17]**	**[1.06–1.28]**	**[1.03–1.18]**	**[1.06–1.28]**	**[1.05–1.23]**
Sex (female)	0.50		0.95		0.50	
	[0.22–1.14]		[0.36–2.52]		[0.17–1.46]	
Systolic blood pressure	1.00		1.00		1.03	
	[0.98–1.03]		[0.97–1.03]		[1.00–1.06]	
Family Hx DM	**3.41**	**2.75**	2.46		2.03	
	**[1.49–7.81]**	**[1.32–5.74]**	[0.87–6.97]		[0.68–6.06]	
Family Hx stroke	1.09		1.78		0.95	
	[0.43–2.73]		[0.62–5.15]		[0.26–3.47]	
MACE	1.64 [ 0.67–4.03]		1.53 [0.50–4.74]		**4.05 [1.25–13.16]**	**3.20 [1.14–8.93]**
c-Statistic	0.748	0.71	0.772	0.69	0.85	0.79

*Model 1 includes all variables, Model 2 includes a step-wise backward selection of variables using p-value threshold of 0.25/0.05 for inclusion/exclusion.

**Bolded values indicate *p* < 0.05, Wald Chi-Square test.

## Discussion

Our study tested the feasibility of developing a model to predict the risk of patients with normal hemoglobin A1c or prediabetes to progress to to diabetes within a four-year period. The vast majority of the patients in the study remained normal hemoglobin A1c or prediabetic (HbA1c 5.7–6.5%) during the timeframe. Among those who progressed to diabetes, we identified several distinct increa sing HbA1c trajectories that were associated with different risk factors. BMI was consistently found to be associated with all increasing HbA1c trajectories. The steady-rising HbA1c trajectory (C2) was associated with baseline age, BMI and having a family history of DM. Although C66 and C12 appeared to have very similar late-rising HbA1c trajectories, their baseline risk factors were distinct. Only BMI was a significant risk factor for C66, whereas age, BMI and family history of MACE were significant risk factors for the C12 trajectory.

Although many studies have analyzed HbA1c trajectories in type-1 diabetes mellitus or after initial diagnosis of T2DM, only a few studies have examined HbA1c trajectories in normoglycemic or prediabetes populations [19]. Heianza et al. reported a late rising HbA1c trajectory in a subset of 193 patients who developed diabetes in a 10-year retrospective analysis of 1722 prediabetic patients [13]. Consistent with these observations, we found two late-rising HbA1c trajectories associated with different risk factors. In addition, we found a late rising HbA1c (C12) trajectory associated with age, BMI and a personal history of MACE as risk factors. In another study, Shearer et al. (2016) examined risk factors associated with HbA1c trajectories in a 12-year prospective study of young adults. They reported three increasing HbA1c trajectories (low, medium and high), where high waist circumference, high waist-height ratio and being a smoker at the age of 26 predicted the least favorable HbA1c trajectory [20]. In contrast, our study focused on older adults and included baseline chronic conditions as potential risk factors. We did not look at the personal history of smoking for our patients but we looked at all significant comorbidities and didn’t find any association of these (except history of MACE) with any of the increasing HbA1c trajectories.

It is well known that HbA1c levels rise with age [13,20–23]. We found that age was a significant risk factor for both the steady-rising HbA1c trajectory (C2) as well as one of the late-rising HbA1c trajectories (C12). The significance of the association between age and rising HbA1c trajectories diminished when other variables were included in the multivariate analysis (Model 1, Table 2). This suggests that other variables may have a complex relationship with age. Indeed, some evidence suggests that age may not be a predictor of diabetes progression in older adults. For example, in a recent prospective study of 71–90 year old adults, it was reported that death or regression to normoglycemia from prediabetes was more frequent than progression to diabetes [23].

Previous work from our group showed that prediabetes is a risk factor for MACE [12]. Research indicates that many of the inflammatory processes and immunological markers that play a role in increasing the risk for CVD in diabetes can also be found in prediabetes as well [24]. Chronic low grade inflammation was reported to be similar in prediabetic and diabetic patients. However, few studies have suggested that MACE may be a risk factor for developing diabetes. To our knowledge, our study findings are the first to suggest that MACE presence is a risk factor for rapid progression to diabetes mellitus. One study looking at patients with non-ST elevation myocardial infarction and multi-vessel disease, who underwent successful implantation of newer-generation drug eluting stent and were prediabetic, reported that they had worse outcomes (cardiac death and overall death) compared to normoglycemic patients and comparable to those with diabetes [25]. However, they did not report on the impact of MI and multi-vessel disease on the progression to diabetes [25]. Normalization of hemoglobin A1c values has been suggested as a goal in patients with prediabetes for decreasing the overall cardiovascular risk and microvascular disease [12]. However, as mentioned above, this strategy may not be fruitful in older populations who are at higher risk of mortality and cardiovascular disease due to other factors rather than progression to diabetes [23]. Therefore preventative strategies should be tailored with respect to the age of the individual as well as their four-year risk of progressing to diabetes.

Data from meta-analysis has demonstrated a cumulative four-year incidence of T2DM from prediabetes of 14% (11–21%) [10]. However, in our study, we observed a four year T2DM incidence of 27.3% from prediabetic ranges of HbA1c. The likely explanation of these differences is that our study was not designed to investigate the natural history of diabetes progression. The intent of our study was to identify risk factors that were associated with a rapid progression to diabetes. Therefore, we selected the cohort based on the highest recorded HbA1c values (index year) for each individual and examined risk factors four years prior to the index year that may be associated with a rise in HbA1c levels.

To prevent the onset of diabetes and its associated complications, including cardiovascular events and disease [16,26], resources can be directed towards diabetes prevention programs focused on weight loss and lifestyle changes in these high risk individuals. The Diabetes Prevention Program Outcomes Study (DPPOS) found that lifestyle intervention and to a lesser extent metformin use significantly reduced the development of diabetes over 15 years in those that were determined to be at high risk of developing diabetes [27]. Similarly, long term follow up of The Da Qing Diabetes Prevention Study found that a six-year lifestyle intervention can reduce the long term risk of diabetes in addition to cardiovascular events and all-cause mortality in individuals with impaired glucose tolerance [28,29]. Not only have these interventions been shown to be effective, but also data from DPPOS found intensive lifestyle intervention to be cost-effective compared to placebo after 10 year follow up [30]. Delaying or preventing the onset of T2DM can delay or prevent costs and health care utilization related to treatment of diabetes and surveillance and management of its associated complications.

### Limitations

Given the retrospective nature of the study design, our data accuracy and completeness are limited to the accuracy of data found in the electronic health records. In addition, the data for this study are from a single health system and may not be generalizable to other populations. Lastly, the cohort was defined based on having three or more HbA1c values over a five-year period, with the highest HbA1c value in the final index year. Therefore, our analysis does not consider how long the patient remained prediabetic prior to the time window used in this study. By selecting subjects with consecutive HbA1c values during a five-year period, we may have biased the study to individuals who are actively participating in preventative care and have healthier lifestyles than the general population.

## Conclusion

The results of our study suggest that the progression to diabetes from normal hemoglobin A1c or prediabetes within four years is associated with baseline BMI. In addition, a steady rise in HbA1c during this time frame is associated with age and family history of T2DM, whereas age and personal history of MACE were associated with a rapid rise in HbA1c. We suggest that perhaps a more aggressive preventative approach should be considered in patients with a family history of T2DM who have high BMI and year-to-year increase in HbA1c, whether they have normal hemoglobin A1c or they have prediabetes.

## Data Availability

The data is available upon request.
